# Plant-Derived Phenolics Inhibit the Accrual of Structurally Characterised Protein and Lipid Oxidative Modifications

**DOI:** 10.1371/journal.pone.0043308

**Published:** 2012-08-29

**Authors:** Arantza Soler-Cantero, Mariona Jové, Daniel Cacabelos, Jordi Boada, Alba Naudí, Maria-Paz Romero, Anna Cassanyé, José C. E. Serrano, Lluis Arola, Josep Valls, Maria Josep Bellmunt, Joan Prat, Reinald Pamplona, Manuel Portero-Otin, Maria-José Motilva

**Affiliations:** 1 Departament of Food Technology, CeRTA-TPV, Escola Tècnica Superior d′ Enginyeria Agrària, Universitat de Lleida, Lleida, Spain; 2 NUTREN-Nutrigenomics Center, Department of Experimental Medicine, Parc Científic i Tecnològic Agroalimentari de Lleida-Universitat de Lleida-IRBLLEIDA, Lleida, Spain; 3 Department of Biochemistry and Biotechnology, Nutrigenomic Research Group, Universitat Rovira i Virgili, Tarragona, Spain; 4 Shirota Functional Foods, S.L., Reus, Spain; University of Valencia, Spain

## Abstract

Epidemiological data suggest that plant-derived phenolics beneficial effects include an inhibition of LDL oxidation. After applying a screening method based on 2,4-dinitrophenyl hydrazine- protein carbonyl reaction to 21 different plant-derived phenolic acids, we selected the most antioxidant ones. Their effect was assessed in 5 different oxidation systems, as well as in other model proteins. Mass-spectrometry was then used, evidencing a heterogeneous effect on the accumulation of the structurally characterized protein carbonyl glutamic and aminoadipic semialdehydes as well as for malondialdehyde-lysine in LDL apoprotein. After TOF based lipidomics, we identified the most abundant differential lipids in Cu^++^-incubated LDL as 1-palmitoyllysophosphatidylcholine and 1-stearoyl-sn-glycero-3-phosphocholine. Most of selected phenolic compounds prevented the accumulation of those phospholipids and the cellular impairment induced by oxidized LDL. Finally, to validate these effects in vivo, we evaluated the effect of the intake of a phenolic-enriched extract in plasma protein and lipid modifications in a well-established model of atherosclerosis (diet-induced hypercholesterolemia in hamsters). This showed that a dietary supplement with a phenolic-enriched extract diminished plasma protein oxidative and lipid damage. Globally, these data show structural basis of antioxidant properties of plant-derived phenolic acids in protein oxidation that may be relevant for the health-promoting effects of its dietary intake.

## Introduction

The oxidative stress hypothesis of atherosclerosis is based on the fact that free radical-derived damage participates in atherogenesis pathophysiology by means of LDL modification, among other mechanisms [1]–[3]. Supporting this hypothesis, LDL modified by oxidation (oxLDL) has been detected in atherosclerotic lesions [4]–[6] and oxLDL exhibits various proatherogenic activities *in vitro* [3], [7].

It is important to emphasize that oxLDL represents a heterogeneous population of modified forms of LDL that differ greatly in their chemical composition and biological properties. The conversion of native LDL into highly modified LDL via oxidative processes can occur by two major mechanisms: In the first case, the events start with the complete loss of LDL's endogenous antioxidants (i.e., α-tocopherol, ubiquinol-10), followed by the conversion of a majority of the polyunsaturated fatty acids (PUFA) into their corresponding hydroperoxides. Then, these primary lipid oxidation products are fragmented into secondary lipid oxidation products, such as malonyldialdehyde or 4-hydroxynonenal, which can react with the N∑-amino group of lysine residues from LDL apoprotein (Apo B-100). Consequently, the particle's electrophoretic mobility increases and the lipoprotein becomes “high uptake” [8]. Additionally, in the subendothelial space, oxLDL may exert a “Trojan horse effect”, e.g. allowing the diffusion of those lipid mediators modifying endothelial and vascular smooth muscle cells. The second case is characterized by the immediate and preferential oxidation of amino acid residues from Apo B-100 in the absence of substantial consumption of lipid soluble antioxidants and/or occurrence of lipid peroxidation [9]. Nevertheless, apoprotein oxidative modifications have been not studied as extensively as lipid-phase ones.

The beneficial effects of Mediterranean diet concerning cardiovascular diseases are well known. Most of them are associated with olive oil and wine consumption [10], [11], nutrients with a high content of phenolic acids. With regard to the influence of diet on atherosclerosis, the ingestion of fruits and vegetables (phenolic-rich vegetal sources) is related to a lesser development of atheroma plaque [12], [13]. This effect is mainly attributed with their protective effects against LDL oxidation [14]–[17]. Despite a great deal of research having been devoted to the prevention of lipid peroxidation in LDL by antioxidants, including phenolic acids [18]–[21], few studies have reported the prevention of protein oxidation in LDL by exogenous antioxidants. Apo B-100 modifications, e.g., the binding of lipid peroxidation products or direct oxidation of amino acid side-chain residues, are thought to finally result in the formation of new epitopes that are specifically recognized by scavenger receptors [22]–[25], but the potential preventive effect of nutritional compounds on apoprotein oxidative modifications have not been studied so extensively.

To fill those gaps, the antioxidant effect of 21 different vegetal-derived phenolic compounds (mainly founded in olive oil and grape-derived products) in the oxidation of apolipoprotein of human LDL was assessed in this study. For that purpose, the quantification of carbonyl groups (detected by Western-Blot) was carried out in LDL-model systems. The antioxidant behaviour of the more active phenols was then further characterized by measuring the protection of the lipidome changes induced by Cu^++^, the accumulation of specific oxidation and lipid peroxidation markers in LDL apoproteins. The preventive role on the loss of cell viability induced by Cu^++^-treated LDL was tested using these phenolic compounds. Finally, in order to test whether those or related phenolic compounds would have similar effects *in vivo* in an atherosclerosis model, carbonyl content of plasma proteins and lipid oxidation markers were analysed in hypercholesterolemic hamsters fed with a phenolic- enriched diet.

## Methods

### Reference Compounds

*α*-Tocopherol was purchased from Sigma-Aldrich Chemical Co (St. Louis, MA, USA). Phenolic standards from the following sources were used without further purification: 2-(3,4-dihydroxyphenyl)-4,5-dihydroxy-3-[3,4,5-trihydroxy-6-[(3,4,5-trihydroxy-6-methyl-oxan-2-yl)oxymethyl]oxan-2-yl]oxy-chromen-7-one (rutin), 4′,5,7-tihydroxyflavone (apigenin), apigenin 7-*O*-glucoside, 3′,4′,5,7-tetrahydroxyflavone (luteolin), luteolin 7-*O*-glucoside, *trans*-4-hydroxycinnamic acid (*p*-coumaric acid), 2-(3,4-dihydroxyphenyl) ethyl alcohol (OH-tyrosol) (3,4-DHPEA), 2-(4-hydroxyphenyl) ethyl alcohol (tyrosol) (*p*-HPEA), oleuropein, verbascoside and vanillin from Extrasynthese (Genay, France); 3,4-dihydroxycinnamic acid (caffeic acid), ferulic acid, and 3,4,5-trihydroxybenzoic acid (gallic acid) from Fluka Co. (Buchs, Switzerland); Pinoresinol from Arbonova Sales (Turku, Finland). Phenolic enriched grape seed extract was obtained as previously described [26].

### Isolation of Phenolic Compounds by Semipreparative HPLC

Secoiridoid derivatives 4-(acetoxyethyl)-1,2-dihydroxybenzene (3,4-DHPEA-AC), 4-hexenoic acid, 4-formyl-3-(2-oxoethyl)-2-(3,4 dihydroxyphenyl) ethyl ester (3,4-DHPEA-EDA), methylated form of the oleuropein aglycone (ME 3,4-DHPEA-EA) and 4-hexenoic acid, 4-formyl-3-(2-oxoethyl) 2-(4-hydroxyphenyl) ethyl ester (*p*-HPEA-EDA) were isolated from virgin olive oil phenolic extract by semi-preparative HPLC method according to the method of Artajo et al. [27]. Stock solutions of commercial standards and phenolic compounds isolated from virgin olive oil were dissolved in methanol/H_2_O (80∶20 v/v) and stored at −40°C before the evaluation of their antioxidant activity. The chemical structures of the phenols included in the study are shown in Figure S1.

### Hypercholesterolemic hamsters and human plasma

Twelve male Gold Syrian hamster weighing 127.55±6.75 g were randomly assigned to two groups (n = 6 for each group) with approximately equal mean group body weights. They were caged and maintained in a 12∶12 (light∶dark) cycle at 22±2°C and 50±10% relative humidity with free access to both food and water. Food intake and body weight were controlled every week.

The control group ate an atherogenic diet [28] in which the cholesterol content had been set at 5% and which was supplemented with 15% of lard, and the experimental group ate this atherogenic diet supplemented with a 0.2% of phenolic-enriched vegetal extract (Table 1). The diets were maintained during 12 weeks and the animals were deprived of food for 15 h. Hamsters were anesthetized and then sacrificed by heart puncture and plasma and serum were collected and stored at −20°C until analysis. For metabolomic analyses, the method of Wikoff et al was used [29]. The plasma triglycerides, cholesterol, HDL, LDL, and alkaline phosphatase content were quantified by enzymatic colorimetric reactions using commercial kits (Spinreact, Girona, Spain).

**Table 1:** Control and supplemented diet composition.

Component	Control (g)	Supplemented (g)
Casein	200	200
L-Cysteine	3	3
Corn starch	362	362
Sugar	140	140
Corn oil	0	0
Cellulose	50	48
Minerals	35	35
Lard	150	150
Vitamins	10	10
Cholesterol	50	50
Polyphenol extract	0	2
**Total weight**	**1000**	**1000**
Energy (Kcal/g diet)	4.62	4.62
**Carbohydrates (g/Kg)**	**502**	**502**
% Energy	43.5	43.5
**Proteins (g/Kg)**	**203**	**203**
% Energy	17.6	17.6
**Lipids**	**200**	**200**
% Energy	39	39

The control diet is an atherogenic diet.

Human plasma was obtained from 6 different, healthy male donors after an overnight fast (mean age: 25±3) by standard venepuncture and centrifugation using EDTA coated Vacutainer tubes. Both plasma obtention and animal experiments were supervised and approved by the Experimental and Ethics Committee of the University of Lleida.

### Protein oxidation screening method: Protein oxidation and Western blot analysis

Aliquots of phenolic acid compounds dissolved in methanol were transferred to Eppendorf tubes and desiccated under a nitrogen current at room temperature. The dried phenols were redissolved to a final concentration of 5 µM with PBS (except when indicated) containing dissolved protein. Then, protein (700 µg/ml) was oxidized by exposure to different prooxidants. We used i) CuSO_4_ (5 µmol/l free Cu^++^); ii) Hemin:H_2_O_2_ (30 µmol/l: 5 µmol/l); iii) H_2_O_2_ (5 µmol/l); iv) Ascorbate:Fe^3+^ (1.5 mmol/l: 8 µmol/l); v) ultraviolet radiation (λ 254 nm) and v) myeloperoxidase (MPO) (0.77 U/mL and H_2_O_2_ 100 µmol/l) in phosphate buffered media at 37°C for 3 hours, according published procedures [30]. All samples were used immediately or stored at −80°C for further analysis.

To assess the extent of protein oxidation, 2,4-dinitrophenylhydrazine (DNP)-reactive carbonyls were measured by Western Blot as previously described [31] (See Methods S1 to further information).

### Measurement of glutamic (GSA) and aminoadipic (AASA) semialdehydes and malondialdehyde lysine (MDAL)

The concentration of chemically characterized markers of protein oxidative modification GSA, AASA, and MDAL, in LDL apoproteins was evaluated by gas chromatography/mass spectrometry (GC/MS) as previously described [31] (See Methods S1 to further information).

### Lipidome analyses

Lipid composition was assessed by both fatty acid analysis (see Methods S1 to further information) and time of flight mass spectrometry (TOF)-based lipid molecular species analyses. In both cases the total lipids from LDL were extracted with chloroform:methanol (2∶1, v/v) in the presence of 0.01% butylated hydroxytoluene as previously described [31].

For TOF-based lipid molecular species analyses, lipid extracts (from LDL) or methanolic extracts (from hamster's plasma) were submitted to mass-spectrometry using a LC ESI-QTOF MS/MS 6520 (Agilent Technologies, Barcelona, Spain), coupled to a capillary LC module using an untargeted approach as described [29] (see Methods S1 to further information). In order to offer a relative quantification of 1-palmitoyl-2-(5-oxovaleryl)-*sn*-glycero-3-phosphocholine (POVPC) and 1-palmitoyl-2-glutaryl-*sn*-glycero-3-phosphocholine (PGPC), bioactive lipids present in oxLDL mass profiles [32] were integrated for an m/z of 594.3 for POVPC and 610.2 for PGPC with a Δ of 0.01 Da.

### FRAP assay

The ferric reducing antioxidant power of the samples was estimated according to the procedure previously described [33], [34]. Briefly, FRAP reagent, was mixed with distilled water and either of sample or appropriate reagent blank. The readings at 30 min were selected for calculation of FRAP values. Reduction power activities were as µmol of Trolox equivalents, per gram of dry matter.

### Cell viability

Both HMEC (kindly providen by Anne Negre-Salvayre, INSERM, Toulouse [35] and HepG2 viability were measured with the MTT-based Cell Toxicity Colorimetric Assay Kit (Sigma-Aldrich, St.Louis, MA, USA) according to the manufacturer's instructions after oxLDL tert-butylhydroperoxide (t-BOOH) as described [36], [37]. The results were expressed as the percentage of viability versus cells exposed to non-oxidized LDL or untreated with t-BOOH. Further details are described in the Methods S1 section.

### Statistical analyses

All statistics were analysed using the SPSS software (SPSS Inc., Chicago, IL, USA). Differences between the groups were analysed by the Student's T tests or ANOVA (with post-hoc analyses for detecting differences between specific pairs), after assessment of normal distribution of variables by the Kolmogorov-Smirnoff test. Correlations between variables were evaluated by the Pearson statistic and plotted with the Metaboanalyst software [38] The 0.05 level was selected as the point of minimal statistical significance in every comparison.

## Results

### Effect of individual phenolic compounds on LDL oxidation. LDL oxidation by different methods induces accumulation of carbonyl in LDL apoproteins: differential inhibitory potential of phenolic compounds

**Figure 1:**
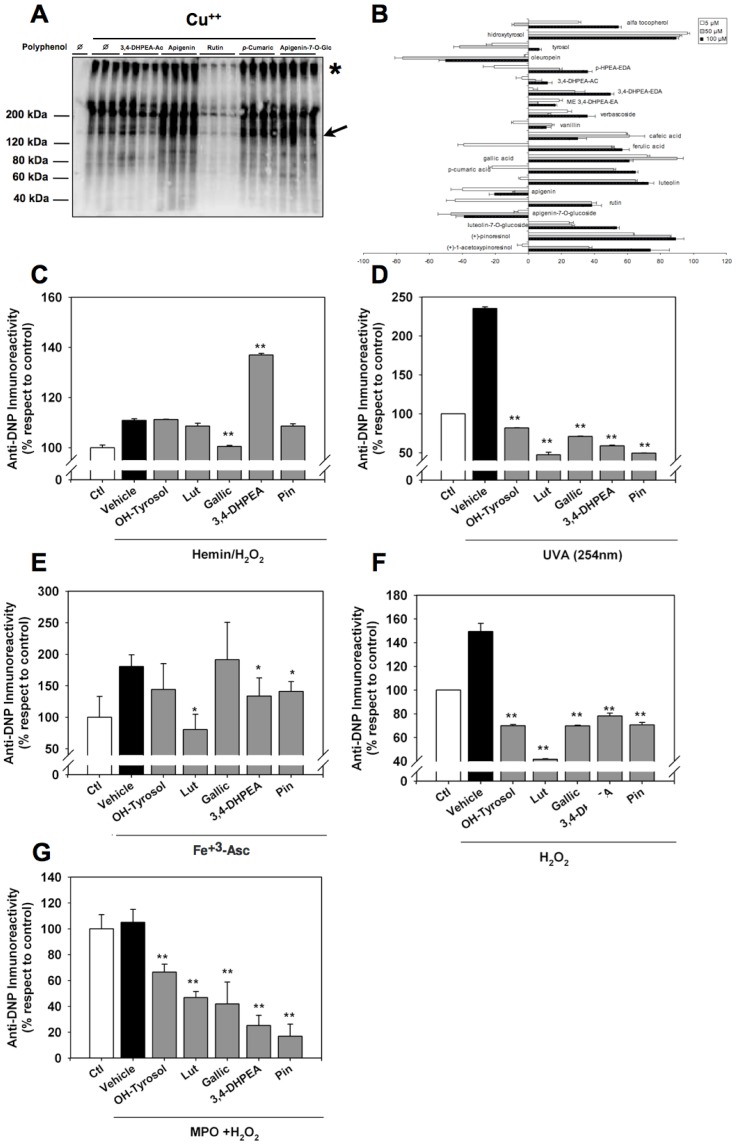
Plant-derived phenolic acids inhibit protein oxidation in isolated human LDL to varying degrees. **A.** Representative Western Blot of the screening phase for LDL apoprotein protection in oxLDL. Data are presented for 3,4-DHPEA-AC, apigenin, rutin, *p*-cumaric acid and apigenin-7-O-glucoside at 100 µM. Veh:Vehicle, * indicates Apo B-100 molecular weight and arrow indicates protein fragmentation induced by oxidation. **B:** Effect of plant-derived phenolic acids in protein carbonylation in Cu^++^-incubated LDL. All phenols were tested at three concentrations (5 µM, 50 µM and 100 µM). LDL and Cu^++^ incubated LDL were used as control of native and oxidized LDL. The antioxidant capacity of each phenol at different concentrations is expressed as percentual values, considering that the immunoreactivity of Cu^++^-incubated LDL is considered 0% of antioxidant capacity - positive values reveal decreased carbonyl formation and negative values reveal increased protein carbonylation-. LDL Apoprotein oxidation was also prevented to various extents in oxidation induced with Hemin:H_2_O_2_ (**C**), Ultraviolet radiation (254 nm) (**D**), Fe^3+^:Ascorbate (**E**), H_2_O_2_ (**F**) and MPO: H_2_O_2_ (**G**) as indicated in the *Methods and Materials* section. In the case of C-G, phenols were tested at 5 µM. In these, carbonyl contents are expressed as percentual values, considering that the immunoreactivity of LDL incubated without oxidant (Ctl) as 100% of carbonyl content. *p<0,05 and ** p<0,01 by ANOVA respect to oxidant-incubated LDL. Data shown are mean±S.D., (n = 4 for each data point).

The antioxidant capacity was quantified after Western Blot of DNP reactive carbonyls in LDL apoproteins (Figure 1A) being the value corresponding to oxLDL considered 0% of antioxidant capacity. The anti-DNP immunoreactivities which are lower than that found in oxLDL were interpreted as anti-oxidant activity and that which are higher as prooxidant activities. The different phenols were tested at three concentrations (5, 50 and 100 µM) (Figure 1B), using α-tocopherol as a reference. Apoprotein oxidation was significantly inhibited by the majority of the phenols tested. The OH-tyrosol showed the maximal efficiency even at 5 µM, higher than the efficiency of α-tocopherol. Luteolin (flavonoid), pinoresinol (lignan), gallic and caffeic acids showed a good efficiency which was concentration-dependent. These phenols reduced the Cu^++^ inducted oxidation by between 60 and 80%. The secoiridoid derivatives (3,4-DHPEA-EDA and *p*-HPEA-EDA) showed a slight activity, similarly to α-tocopherol. Other phenols, such as verbascoside, vanillin, 3,4-DHPEA-AC and the methylated form of the oleuropein aglycone (ME 3,4-DHPEA-EA) showed lower antioxidant activity with oxidation inhibition values below 20%. The prooxidant effect shown by some phenols, such as oleuropein, tyrosol and apigenin in its aglycone and glucosidic forms should also be noted.

As Figure 1B shows, different phenolic compounds exhibit differential effects in a dose dependent fashion, in the apoprotein moiety of human LDL. According to this first screening, the more active phenols selected were: OH-tyrosol (OH-tyrosol), 3,4-DHPEA-EDA (3,4-DHPEA) as secoiridoid derivative; pinoresinol (Pin) as a lignane; luteolin (Lut) as a flavonoid; and gallic (Gallic) acids as a phenolic acid.

In order to extend those findings from Cu^++^ to other oxidation systems, we measured the effects of phenolics in different systems ranging from ultraviolet radiation to enzymatic paradigm (Figure 1C, D, E, F and G). Of those, only UVA, Fe^+3^-Asc and H_2_O_2_ increased carbonyl content more than 50% in LDL (Figure 1D, E and F). In those systems, all phenols analysed had a strong antioxidant action, with the exception of Fe^+3^-Asc, where only luteolin (and 3,4-DHPEA and pinorresinol to a minor extent) had a significant effect. Of note, pathogenically relevant systems such as MPO+H_2_O_2_ do not offer a significant increase in carbonyl staining (Figure 1G). Most importantly, all selected phenolic compounds are able to significantly inhibit the accrual of carbonyl modification after MPO+H_2_O_2_ incubation, being pinoresinol the most active.

In order to extent those results to other proteic systems, BSA and human plasma were oxidized using Hemin-H_2_O_2_, UVA and Fe^3+^-Asc. These results (Figure S2) do not allow to distinguish any individual phenol as a general antioxidant, i.e. its effect being independent of the protein and oxidation-system used. Generally, BSA is less oxidizable than LDL in same conditions, and only pinoresinol and luteolin diminish significantly protein oxidation in specific systems (Figure S2B and S2C). In contrast, human plasma is more oxidizable than BSA (under UVA and Fe^+3^-Asc). Under Fe^3+^-Asc, again pinoresinol and luteolin stand out as significant inhibitors of protein oxidation in plasma (Figure S2E and S2F). Of note, several polyphenols (notably gallic acid) exhibited prooxidant actions under these conditions (Figure S2B and S2D).

### Cu^++^ incubation induces accumulation of metal-catalyzed oxidation (MCO), lipoxidation markers in LDL apoproteins and changes in LDL lipidome: differential inhibitory potential of phenolic acid compounds

**Figure 2:**
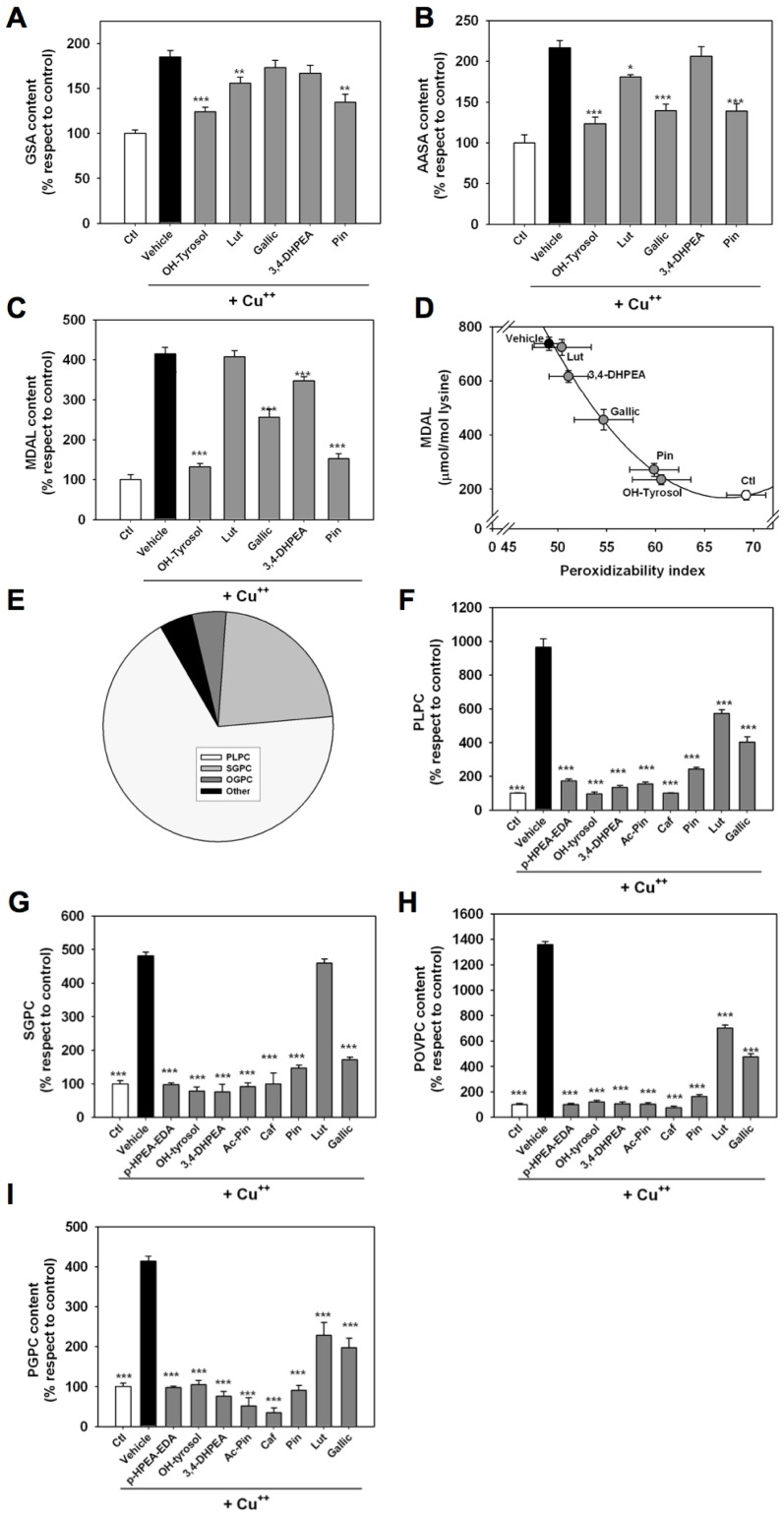
Cu^++^-incubated LDL show significant increases in the amounts of mass-spectrometry characterized protein and lipid oxidation markers, whose formation is inhibited by selected plant-derived phenolic acids. **A–C** show GC/MS analyses of GSA and AASA (markers of metal-catalyzed oxidation), and MDAL, originated from lipoxidation. Values shown are % changes of mean ± S.E.M. over values from non-oxidized LDL (GSA: 5945±61 µmol/mol lysine; AASA: 1128±20 µmol/mol lysine; and MDAL: 177±23 µmol/mol lysine); *p<0,05; ** p<0,01 and *** p<0,001 respect to Cu^++^ incubated LDL. D: protein lipoxidative damage shows a quadratic relationship with peroxidizability index (PI) (r^2^ = 0,911;p<0,0001; model:[MDAL] = 1,03*[PI]^2^-151,26*[PI]+5698) **E:** Pie plot showing distribution of differential (Student's T test p<0.001) lipid species between control and Cu^++^-incubated LDL, after TOF-based lipidome analyses. **F:** Effect of plant-derived phenolic acids in the accumulation of PLPC induced by Cu^++^-incubation. **G:** Effect of plant-derived phenolic acids in the accumulation of SGPC induced by Cu^++^-incubation. **H:** Effect of plant-derived phenolic acids in the accumulation of POVPC induced by Cu^++^-incubation. **I:** Effect of plant-derived phenolic acids in the accumulation of PGPC induced by Cu^++^-incubation. Values shown are mean ± S.E.M. over values from Cu^++^-incubated LDL, *** p<0,001 (n = 4 for each data point).

Taking into account that DNP reactive carbonyls could arise from either lipid peroxidative damage or the direct modification of aminoacid residues by MCO [39] specific probes for each of those oxidative modifications were measured by using GC/MS. The results show that Cu^++^ incubation led to significant increases in the MCO markers GSA, AASA and an even greater increase in the lipoxidation marker MDAL (Figure 2A, B and C). OH-tyrosol and the lignane were the most effective compounds for inhibiting GSA accumulation. Luteolin also prevented its accumulation in Cu^++^-treated LDL. Neither gallic acid, nor 3,4-DHPEA-EDA were effective (Figure 2A). A similar pattern was observed for AASA accumulation, but in this case, gallic acid was significant inhibitors of its formation (Figure 2B). Finally, OH-tyrosol and pinoresinol were potent antioxidants in considering MDAL accumulation, while 3,4-DHPEA-EDA, and specially luteolin (with no significant effect), were among the lowest in this sense (Figure 2C). To reinforce the importance of the lipid composition in relation with the lipoxidative modifications of proteins, a significant correlation was observed (r^2^ = 0.91; p<0.0001) between PI and MDAL formation (Figure 2D).

Carbonyl modification of apoproteins can arise from lipid peroxidation. In such a case, the LDL fatty acid composition exhibits PUFA consumption. To test this, the fatty acid composition of LDL was analysed after Cu^++^-induced oxidation, demonstrating significant losses in PI and PUFA content (Table 2). For this reason we examined the potential for prevention of this phenomenon. OH-tyrosol, and the lignane were among the most potent compounds, almost preventing the effect of Cu^++^. Gallic acid was less potent, and luteolin and 3,4-DHPEA-EDA had almost no effect on the oxidative modification of LDL

**Table 2:** Effect of Cu^++^ and phenolic acids in LDL fatty acid composition.

	Ctl	Cu^++^	OH-Tyrosol	Lut	Gall	3,4-DHPEA	Pin
**16:0**	21,11±0,59*	24,78±0,78	23,39±0,84	26,55±0,91	24,25±0,65	24,77±0,8	23,32±0,10
**16:1n-7**	3,06±0,28*	3,27±0,55	3,00±0,66*	3,33±0,26	3,25±0,87	3,10±0,7	3,26±0,15
**18:0**	11,55±0,16	11,08±0,93	8,90±0,30*	11,63±0,35	10,08±0,41	11,41±0,0	9,36±0,46*
**18:1n-9**	22,77±0,97**	26,38±0,26	24,91±0,27	25,37±0,06	25,24±0,93	25,74±0,0	24,61±0,69
**18:2n-6**	28,84±0,50**	24,97±0,21	30,39±0,33**	23,11±0,81	28,02±0,8**	26,09±0,9*	29,78±0,13**
**20:3n-6**	1,59±0,47*	0,98±0,07	1,39±0,85*	0,88±0,02	1,18±0,14*	1,01±0,1	1,24±0,22*
**20:4n-6**	6,05±0,96**	3,08±0,70	4,62±0,19*	2,67±0,25	3,77±0,14*	3,32±0,6	4,29±0,65*
**ACL**	17,74±0,9	17,5±0,7	17,6±0,2	17,5±0,1	17,5±0,8	17,5±0,7	17,6±0,1
**SFA**	34,41±1,2*	38,1±0,3	33,8±0,0*	40,2±0,6*	36,1±0,4	37,9±0,8	34,6±0,3*
**UFA**	65,59±2,2*	61,8±1,7	66,2±1,0*	59,7±0,4	63,8±0,6	62,0±2,2	65,4±1,2*
**MUFA**	26,07±0,7*	30,3±0,5	28,0±0,2*	28,8±0,8	28,7±0,1	29,0±0,2	28,0±0,2*
**PUFA**	39,52±0,8**	31,5±0,2	38,2±0,1**	30,8±0,6	35,1±0,6*	33,0±0,0	37,4±0,2*
**PUFAn-3**	1,67±0,02*	0,9±0,08	1,2±0,1	0,8±0,1	1,0±0,5	1,0±0,7	1,3±0,1
**PUFAn-6**	37,85±1,2*	30,5±0,4	36,9±0,9*	30,05±0,1	34,1±0,1*	31,9±0,3	36,1±0,1*
**DBI**	124,58±3,2**	104,6±0,6	119,0±0,6**	103,2±0,0	111,6±0,8*	106,6±0,5	117,3±0,7**
**PI**	69,26±2,3**	49,1±0,1	60,6±0,1**	50,4±0,2	54,6±0,9*	51,1±0,1	59,8±0,6*

Values: mean±S.E. ACL, average chain length; SFA, saturated fatty acids; UFA, unsaturated fatty acids; PUFA n-6/n-3, polyunsaturated fatty acids n-6 or n-3 series; MUFA, monounsaturated fatty acids; DBI, double bond index; PI, peroxidizability index,

* p<0,05 and ** p<0,01 respect to values in Cu^++^ incubated LDL by ANOVA post-hoc analyses (n = 4 for each data point).

After TOF-based analyses of oxLDL, palmitoyllysophosphatidylcholine (PLPC), 1-stearoyl-sn-glycero-3-phosphocholine (SGPC) and 1-oleoylglycerophosphocholine (OGPC) comprised more than 90% of the differentially present lipids in Cu^++^-treated LDL (Figure 2E). Similarly to the fatty acid analyses, luteolin and gallic acid exhibit the lowest capacity for preventing the build-up of PLPC, while the other compounds prevented the accumulation of this compound almost completely. Luteolin was the only compound unable to inhibit the formation of SGPC (Figure 2F and 2G).

Taking into account that 1-palmitoyl-2-(5-oxovaleryl)-sn-glycero-3-phosphocholine (POVPC) and 1-palmitoyl-2-glutaryl-sn-glycero-3-phosphocholine (PGPC) have been identified as biological effectors of oxLDL [32], the presence of those compounds was ensured. As expected, their level increased in Cu^++^-treated samples (12 and 4-fold over untreated samples, respectively). Luteolin and gallic acid prevented the accumulation PLPC to a lower extent when compared with the other compounds (Figure 2F).

### Biological relevance of plant-derived phenolics antioxidant effects: Loss of cell viability induced by Cu^++^-treated LDL and in vivo evidences of protein and lipid antioxidant activity

**Figure 3:**
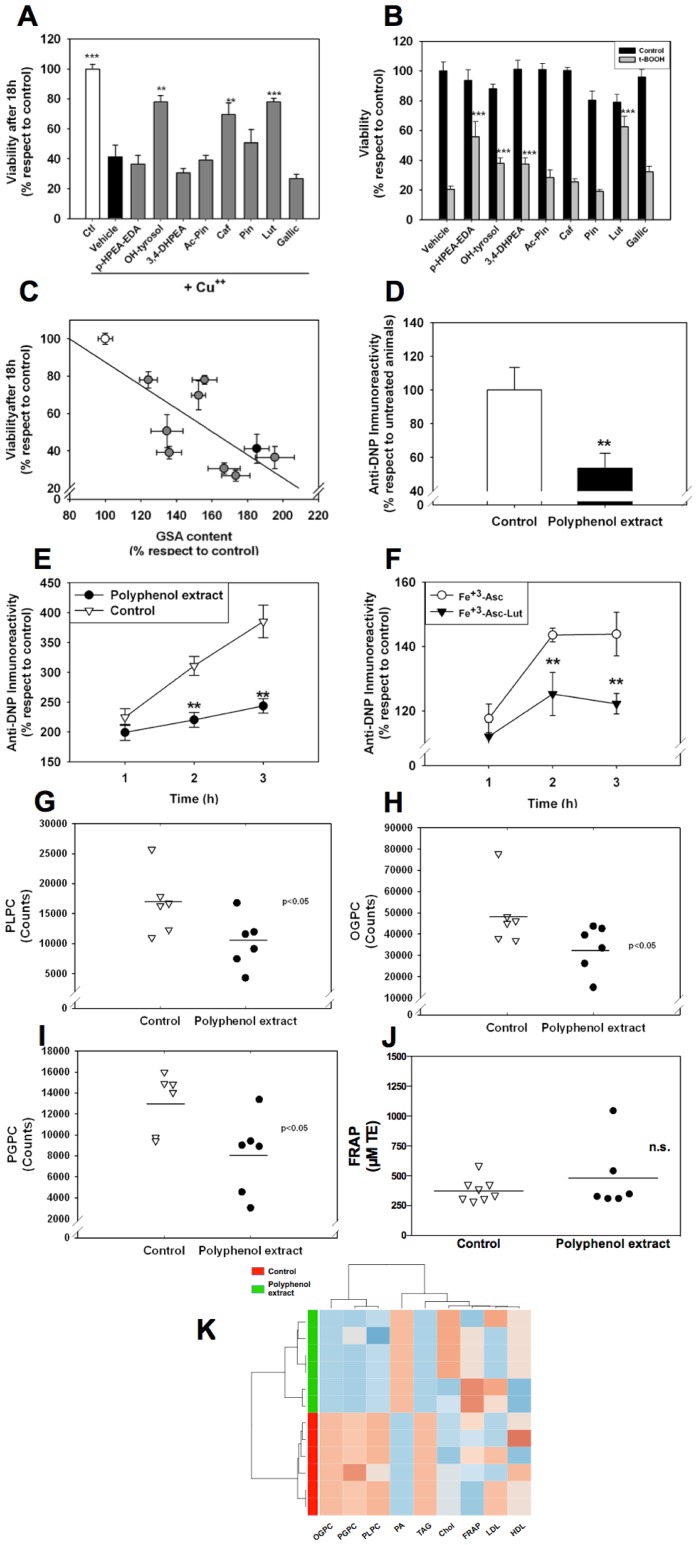
Plant-derived phenolic acids inhibit both the effects of oxidative stress in endothelial and HepG2 cell culture and protein oxidation in diet-induced hypercholesterolemia in vivo. **A:** Effect of plant-derived phenolic acids in cell death in HMEC (endothelial cell line) induced by Cu^++^-incubated LDL; ** p<0,01 and *** p<0,001 respect to cell death induced by Cu^++^ incubated LDL (n = 6 for each data point). Values shown are mean ± S.E.M. over values from samples treated with control LDL **B:** Effect of plant-derived phenolic acids in HepG2 capacity to withstand t-BOOH induced cell death; *** p<0,001 respect to cell death induced by t-BOOH (n = 6 for each data point). Values shown are % changes of mean ± S.E. over values from control samples. **C:** Endothelial cell death induced by Cu^++^-incubated LDL is strongly correlated with GSA content (r = −0.734; p<0,016 by the Pearson correlation test). To validate this in vivo, proteins from hypercholesterolemic hamsters were analyzed for carbonyl content (**D**). Both plasma from hypercholesterolemic hamsters with a dietary supplement of phenolic acid-enriched vegetal extract (**E**) and pooled human plasma (**F**) were oxidized with Fe^3+^-Asc as indicated in the *Methods and Material* section. Aliquots were taken at indicated time points and processed for determination of carbonyl content. **G:** Effect of phenolic acids-enriched diet intake in the steady state levels of a compound compatible with PLPC in plasma from hypercholesterolemic hamsters. **H:** Effect of phenolic acids-enriched diet intake in the steady state levels of a compound compatible with OGPC in plasma from hypercholesterolemic hamsters. **I:** Effect of phenolic acids-enriched diet intake in the steady state levels of a compound compatible with PGPC in plasma from hypercholesterolemic hamsters. J: Effect of phenolic acids-enriched diet intake in FRAP activity of plasma. K: Heat-map showing that phenolic acids-enriched diet induces a significant hierarchical clustering of measured parameters, including both oxidative damage and metabolic (cholesterol, HDL and LDL-cholesterol, triacilglyceride and alkaline phosphatase activities), revealing an important biological effect. The carbonyl content is expressed as a percentual value, considering that the immunoreactivity of plasma from hypercholesterolemic hamsters individuals (Controls in **D**) or protein incubated without oxidant (Ctl) (in **E**) as 100% of carbonyl content. ** p<0,01 by Student's T test respect to untreated animals (***E,G,H,I,J***) or oxidant-incubated human plasma (**F**). Data shown are mean±S.E.M., (n = 4 for each data point). For clustering analyses, values of samples were log transformed and hierarchical clustering was performed using Pearson correlation coefficient as distance measure and the Ward's clustering algorithm. Colors in K express intensity/abundance with red colors showing high levels and blue colors lower levels.

To further reinforce the biological relevance of the antioxidant potential of those compounds and the methodology described here for its identification, we examined the cytotoxic potential of Cu^++^ incubated LDL in an endothelial cell culture model. For this purpose, the endothelial cell line HMEC-1 was treated with Cu^++^-treated LDL and 18 h later, the viability of the cultures was assessed with the MTT assay. The results demonstrate that oxLDL led to a loss of 60% of viability and OH-tyrosol prevented partially those effects (Figure 3A), inducing only a 10% of viability loss. Unexpectedly, luteolin, a compound with a low antioxidant potential, based on lipidome changes, showed a significant preventive effect on the oxLDL induced loss of viability.

To ascertain whether this was due to a specific cellular effect (e.g. by modulation of antioxidant cellular responses in endothelial cells), we examined the potential influence of those compounds in *tert*-BOOH-mediated cell death in a HepG2 cell line, an unrelated cell line. Those analyses demonstrate that luteolin, OH-tyrosol and 3,4-DHPEA-EDA were the only compounds able to prevent significantly the loss of viability secondary to treatment with *tert*-BOOH (Figure 3B).

The correlation of cell viability with antioxidant capacity reinforced the pathogenic importance of LDL apoprotein oxidative modification, as that GSA amount showed the most significant correlation with the loss of viability induced by oxLDL (Figure 3C).

Protein antioxidant activity of phenolic acids should be bioavailable. For this reason we analysed the plasma proteins from a hypercholesterolemic model (Golden Syrian Hamsters under hypercholesterolemic diet, showing an increase around 125% in cholesterol levels respect animals under standard chow) under a diet enriched in vegetable-derived phenols. The polyphenol supplementation did not induce any signs of toxicity nor changes in alkaline phosphatase, indicating that it was well tolerated. Despite an effect in LDL cholesterolemia was noted, no significant differences were evidenced in other values analysed (Table S1). Nevertheless, the results (Figure 3D) demonstrate that phenolic-enriched diet significantly diminishes steady state levels of circulating protein carbonyls. Furthermore, their intake reduces oxidability of proteins *ex vivo* under Fe^3+-^Asc system, both in hypercholesterolemic hamsters (Figure 3E) and in human plasma with exogenously added luteolin at 5 µM (Figure 3F). Noteworthy, compounds with masses compatible with oxidized phospholipids as PLPC, OGPC and PGPC were detected in the methanolic extract of hamster plasma (Figure S3, S4, S5). The levels of those metabolites were diminished in animals under phenolic-enriched diet (Figure 3G, H and I). Reinforcing the importance of specificity of oxidative damage measurements, FRAP assay of plasma demonstrated that polyphenol-enriched diet did not change antioxidant capacity (Figure 3J), although it changed significantly profiles of metabolic parameters by hierarchical clustering (Figure 3K).

## Discussion

The present work demonstrates the antioxidant properties of selected plant-derived phenols (mainly found in olive oil and grape-derived products) in protein oxidation both *in vivo* and *in vitro*, and the existence of some heterogeneity in the mechanisms. We offer a novel approach for analysing and screening the antioxidant properties of natural products over LDL modification, especially in its protein components. After a screening phase, five different compounds were selected for further analysis. We have demonstrated for the first time, the effect of those nutritional compounds on the accrual of structurally characterized markers of both protein (GSA, AASA and MDAL) and lipid peroxidation (POVPC, PGPC) in oxLDL. The pathogenic importance of LDL protein oxidation is shown by the fact that it was the only factor that correlated with oxLDL-induced cell death. To extend the validity to an *in vivo* setting, we have shown that phenolic intake in a hypercholesterolemic context diminishes protein carbonyl content in plasma as well as the concentration of lipid peroxidation markers.

In order to establish whether structural features determine the effect on the resistance of LDL to Cu^++^-induced oxidation, the chemical structures of the phenolic compounds should be considered (Figure S1 **)**. Important differences in the protection of the apoprotein oxidation were observed in this study, similar to those observed by other authors using the lag time of LDL as marker oxidation [40], [41]. The differences between those phenols are attributable to their chemical structure, e.g. OH-tyrosol possesses a 3,4-dihydroxy structure linked to an aromatic ring that confers the moiety with a higher proton dislocation, facilitating a higher scavenging activity than observed with tyrosol, which only has a hydroxyl group linked to an aromatic ring (Figure S1). The protective effect of the OH-tyrosol structure has biological relevance as OH-tyrosol and tyrosol are the more simple phenols detected in plasma and LDL after the ingestion of virgin olive oil and red wine [42], [43]. It should be noted that OH-tyrosol, with the highest potential for inhibition of MDAL, has been previously described as a potent inducer of cellular antioxidant responses, both in oxLDL-induced and in tert-BOOH-induced cell stress [36], [44].

The antioxidant capacity shown by oleuropein and its secoiridoid derivatives was quite different. The aglicon derivative of oleuropein (3,4-DHPEA-EDA) and the ligstroside derivative (*p-*HPEA-EDA) showed a positive result as antioxidants, mainly at the maximum concentration tested. These compounds exhibit a high selectivity for lipid-related changes since they mainly prevented Cu^++^ induced changes in lipidome and MDAL in LDL. Although no previous results were available on lipid or protein antioxidant properties, 3,4-DHPEA-EDA is a compound of special interest because of its presence as one of the major phenolic antioxidant compounds in virgin olive oil. Moreover, it is an important source of OH-tyrosol in plasma. In contrast with these data, a similar phenolic structure, the 3,4-DHPEA-AC showed no significant antioxidant activity in this system. This may be attributed to the potential proton dislocation due to double bonds in the elenolic structure in 3,4-DHPEA-EDA. Reinforcing the importance of conjugation, oleuropein, or glucose-conjugated elenolic acid, showed the more remarkable prooxidant activity of all phenols included in the study in agreement with its previous reported pro-oxidant activities [45], [46]. Most interestingly, as oleuropein is considered a proapoptotic agent with potential use as an anti-tumoral agent [47], [48], it may be suggested that those protein prooxidant properties could be involved in the beneficial effects of oleuropein.

Flavonoids such as rutin, flavones (apigenin and luteolin), and their glucosides (apigenin-7-*O*-glucoside and luteolin-7-*O*-glucoside) can scavenge reactive oxygen radicals, by donating a hydrogen atom or electron [49], [50]. Their radical scavenging activity seems to be substantially dependent on three structural groups: (i) the *orto*-dihydroxyl structure (catechol structure) in the B ring, which is the obvious radical target site, (ii) the 2,3 double bond in conjunction with 4-oxo function, which is responsible for electron delocalization, and (iii) the additional presence of both the 3- and the 5-OH groups for maximal radical scavenging potential and the strongest radical absorption. Our data suggest the importance of the *ortho*-dihydroxyl structure in the prevention of protein modification, as luteolin and rutin -both containing this structure- act as more effective antioxidants than apigenin, a very similar molecule. Comparing the antioxidant activity in the copper-induced LDL oxidation of the luteolin and luteolin-7-*O*-glucoside, the antioxidant capacity of both structures was similar. Despite the described preventive effects of those flavonoids in the diminution of the lag-phase of LDL induced by Cu^++^ and other oxidants [51], [52] no data were available on their role as inhibitors of protein oxidation markers. In fact, luteolin can be classified as an agent with more potential for preventing direct oxidation (i.e. GSA and AASA accumulation) than for preventing lipid peroxidation, as it shows a lower power for protecting against Cu^++^-induced lipidome changes in LDL, in agreement with previous data showing a modest inhibition in the formation of thiobarbituric acid-reactive substances driven by Cu^++^ [53]. In contrast with this relative low *in vitro* potential, it shows a high efficiency in both cell systems as it is able to completely block tert-BOOH-induced changes in cell viability and it shows a high potential against oxLDL-induced cell death, in agreement with the reported inhibitory effect of luteolin in oxidized LDL-induced endothelial monocyte adhesion and/or oxidised LDL uptake [53].

Other major phenols quantified in virgin olive oils and grape-derived products are lignans, with a 2,3-dibenzylbutane skeleton, whose concentration is related to the olive cultivar of origin [54]. Although they are important as sources of lignans enterodiol and enterolactone by colonic flora metabolism, our data reveal that acetoxypinoresinol and pinoresinol possess antioxidant activity, in agreement with previous reports, where it was shown that some vegetable extracts, rich in pinoresinol, were able to inhibit LDL oxidation [55], [56]. Their antioxidant activity could be more closely related to their chelating properties than scavenging activity, as they only exhibit a hydroxyl group linked to an aromatic ring. Globally, acetoxypinoresinol and pinoresinol exhibit similar potencies in the inhibition of direct protein oxidation and lipid peroxidative damage, as well as in the prevention of lipidome changes induced by Cu^++^.

With regard to the phenolic acid group (ferulic, cumaric, caffeic and gallic acids) (Figure S1), all the phenols showed antioxidant capacity at 50 and 100 µM. Ferulic and *p*-cumaric acids showed a slight prooxidant activity at 5 µM, that may be attributed to H_2_O_2_ *in vitro* production from phenolic compounds [57], [58]. According to their mechanism of action, the phenolic acids may be classified as free radical terminators interfering with lipid oxidation by rapid donation of a hydrogen atom to peroxy radicals. Their antioxidant activity is related to the molecule containing at least two neighbouring phenolic hydroxyl groups; three such groups are even more desirable facilitating the interference. Results of the present study showed that the caffeic (with two neighbouring phenolic hydroxyl groups) and gallic acids (three hydroxyl groups) shown to have the highest antioxidant capacity. Previous results have demonstrated the antioxidant capacity of gallic and caffeic acids on lipid peroxidation [59]–[61], but only one reported the effect of gallic acids on protein modification, and that was related to nitrosative stress [62]. Concerning their cellular effects, gallic and caffeic acid differed in their effects: while gallic acid was unable to prevent oxLDL-induced or tBOOH-induced loss of viability, caffeic acid inhibited the toxic effects of oxLDL (data not shown). This agrees with the known effect of gallic acid as a proapoptotic agent [63]–[65] and the reported protective effect of caffeic acid in endothelial cell survival after oxLDL treatment [66].

All the phenols studied showed antioxidant capacity in the LDL model, the OH-tyrosol being the more effective. In general, all the phenols showed higher antioxidant activity than α-tocopherol, which could be attributed to the hydrophilic nature of the phenolic structures. In the biphasic microenvironment constituting core lipids and water phase, such as biomembranes and plasma lipoproteins, the location of phenols should be taken into account for understanding their antioxidant activity. Vitamin E (α-tocopherol) seems to be located within the membrane lipids or lipoprotein particles because of its high lipophilicity. However, it is demonstrated that flavonoid aglycones interact in the polar surface region of the phospholipids bilayers in membranes [67], offering a higher protection. This is specially relevant assuming that the antioxidant profile is completely dependent on the system. As shown by experiments with BSA and human plasma, phenolic interaction in protein oxidation is a very complex phenomenon, which depends on not only of the system used for oxidation, but also on the protein, the presence of lipids and the several mechanisms between protein-phenol and free radical source.

In summary, these data show novel antioxidant properties of plant-derived phenolic acid compounds in LDL oxidation and demonstrates phenolic activity in protein oxidation *in vivo*. It is also demonstrated that *in vitro* antioxidant measurements could only partially predict biologic responses to oxidized LDL, thus reinforcing the importance of a multidisciplinary approach for the description of oxidative phenomena in atherosclerosis pathogenesis and its dietary modulation.

## Supporting Information

Figure S1 **Structures of plant-derived phenolics used in the present study.**
(DOCX)

Figure S2 **Representative phenolic acids inhibit protein carbonylation in different oxidative systems up to various extents.** All phenols were tested at 5 µM. Oxidation of BSA (**A,B,C**) or a human plasma pool (**D,E,F**) was induced with Hemin:H_2_O_2_ (**A, D**), Ultraviolet radiation (λ 254 nm) (**B,E**), and Fe^3+^:Ascorbate (**C,F**) as indicated in the *Methods and Materials* section. Carbonyl contents are expressed as percentual values, considering that the immunoreactivity of protein incubated without oxidant (Ctl) as 100% of carbonyl content. ** p<0,01 by ANOVA respect to oxidant-incubated protein. Data shown are mean±S.D., (n = 4 for each data point).
(DOCX)

Figure S3 **Chromatographic and mass-spectra evidence for the presence of PLPC in methanolic extracts of plasma from hypercholesterolemic hamsters.** **A:** Extracted ion chromatogram of m/z 496.3398 (magnified in **B**) showing the peak quantified as PLPC (arrow in **A**). **C:** Mass spectra of the peak quantified as PLPC (arrowhead showing m/z peak with Δ<0.05 Da in comparison to a theoretical mass of C_24_H_50_NO_7_P)
(DOCX)

Figure S4 **Chromatographic and mass-spectra evidence for the presence of OGPC in methanolic extracts of plasma from hypercholesterolemic hamsters.** **A**: Extracted ion chromatogram of m/z 522.3554 (magnified in **B**) showing the peak quantified as OGPC (arrow in **A**). **C:** Mass spectra of the peak quantified as OGPC (arrowhead showing m/z peak with Δ<0.02 Da in comparison to a theoretical mass of C_26_H_52_NO_7_P)
(DOCX)

Figure S5 **Chromatographic and mass-spectra evidence for the presence of PGPC in methanolic extracts of plasma from hypercholesterolemic hamsters.** **A:** Extracted ion chromatogram of m/z 610.3715 (magnified in **B**) showing the peak quantified as PGPC (arrow in **A**). **C:** Mass spectra of the peak quantified as PGPC (arrowhead showing m/z peak with Δ<0.01 Da in comparison to theoretical mass of C_29_H_56_NO_10_P)
(DOCX)

Methods S1 **Supplementary methods.**
(DOCX)

Table S1 (DOCX)
