# Supplementary material for: Plant-Derived Phenolics Inhibit the Accrual of Structurally Characterised Protein and Lipid Oxidative Modifications
Source: PLoS One. 2012 Aug 29;7(8):e43308. doi: 10.1371/journal.pone.0043308 (PMC3430685; doi:10.1371/journal.pone.0043308)
Supplement: Methods S1 — Supplementary methods. (DOCX) [file pone.0043308.s006.docx]

SUPPLEMENTAL ONLINE MATERIAL

Supplemental Methods

*Protein oxidation screening method: Protein oxidation and Western blot analysis*.

Briefly, to 15 µl of sample SDS was added to a final concentration of 6%, and, after boiling for 3 min, 20 µl of 10 mM DNP in 10% trifluoroacetic acid were added. After 7 min at room temperature, 20 µl of a solution containing 2M Tris base, 30% glycerol and 15% β-mercaptoethanol was added for neutralization and sample preparation for loading onto 4-20% gradient pre-made SDS-PAGE gels (Invitrogen, Barcelona, Spain). For immunodetection after SDS-PAGE, proteins were transferred using a Mini Trans-Blot Transfer Cell (BioRad Laboratories, Barcelona, Spain) to PVDF membranes (Immobilon-P Millipore, Bedford, MA, USA). Immunodetection was performed using a rabbit anti-DNP antiserum as the primary antibody (1:4000, Dako, Carpenteria, CA, USA). Peroxidase-coupled secondary antibodies were used from the Tropix chemiluminiscence kit (Tropix Inc, Bedford, MA, USA). Luminiscence was recorded and quantified in Chemidoc equipment (BioRad, Barcelona, Spain), using the Quantity-one analysis software (Biorad). The average mode of background subtraction was chosen to quantify. Control experiments showed that omission of derivatization step, primary or secondary antibody addition produced blots with no detectable signal (data not shown). After developing, the blots were silver-stained as described to check the protein load (1).

*Measurement of glutamic (GSA) and aminoadipic (AASA) semialdehydes and malondialdehyde lysine (MDAL*).

Samples containing 0.4 mg of protein were extensively delipidated using chloroform:methanol extraction (2:1 v/v, 3x) in the presence of 0.01% butylated hydroxytoluene, and the proteins were precipitated by adding 10% of trichloroacetic acid (final concentration) and subsequent centrifugation. The protein samples were reduced overnight with 500 mM NaBH4 (final concentration) in 0.2M borate buffer, pH 9.2, containing 1 drop of hexanol as an anti-foam reagent. The proteins were then reprecipitated by adding 1 ml of 20% trichloroacetic acid and then centrifuging this. The following isotopically labelled internal standards were then added: [2H8]Lysine (d8-Lys; CDN Isotopes); and [2H8]MDAL (d8-MDAL), [2H5] 5-hydroxy-2-aminovaleric acid (for GSA quantization) and [2H4]6-hydroxy-2-aminocaproic acid (for AASA quantization) as described (1). The samples were hydrolysed at 155º C for 30 min in 1ml of 6N HCl, and then vacuum dried. The N,O-trifluoroacetyl methyl ester derivatives of the protein hydrolysate were prepared as previously described (1). The GC/MS analyses were carried out on a Hewlett-Packard model 6890 gas chromatograph equipped with a 30m HP-5MS capillary column (30m x 0.25mm x 0.25 μm) coupled to a Hewlett-Packard model 5973A mass selective detector (Agilent Technologies, Barcelona, Spain). The injection port was maintained at 275º C; the temperature program was 5 min at 110º C, then rising by 2º C/min to 150º C, then by 5º C/min to 240º C, then 25º C/min to 300º C, and finally hold at 300º C for 5 min. Quantification was performed by external standardization using standard curves constructed from mixtures of deuterated and non-deuterated standards. The analytes were detected by selected ion-monitoring GC/MS. The ions used were: lysine and d8-lysine, m/z 180 and 187, respectively; 5-hydroxy-2-aminovaleric acid and d5-5-hydroxy-2-aminovaleric acid (stable derivatives of GSA), m/z 280 and 285, respectively; 6-hydroxy-2-aminocaproic acid and d4-6-hydroxy-2-aminocaproic acid (stable derivatives of AASA), m/z 294 and 298, respectively; and MDAL and d8-MDAL, m/z 474 and 482, respectively. The amounts of products were expressed as the ratio μmol GSA, AASA, or MDAL/mol lysine.

*Lipidome analyses.*

Fatty acid profiles

For fatty acid analyses, the chloroform phase was evaporated under nitrogen, and the fatty acids were transesterified by incubation in 2.5 ml of 5% methanolic HCl for 90 min at 75º C. The resulting fatty acid methyl esters were extracted by adding 2.5 ml of n-pentane and 1 ml of saturated NaCl solution. The n-pentane phase was separated, evaporated under nitrogen, redissolved in 75 μl of hexane and 1 μl was used for the GC/MS analysis. Separation was performed in a SP2330 capillary column (30m x 0.25mm x 0.20μm) in a Hewlett Packard 6890 Series II gas chromatograph (Agilent Technologies, Barcelona, Spain). A Hewlett Packard 5973A mass spectrometer (Agilent Technologies, Barcelona, Spain) was used as the detector in the electron-impact mode. The injection port was maintained at 220º C, and the detector at 250º C; the temperature program was 2 min at 100º C, then rising by 10º C/min to 200º C, then 5º C/min to 240º C, and finally held at 240º C for 10 min. Identification of fatty acid methyl esters was done by comparison with authentic standards and based on mass spectra. Results are expressed as mol %. The following indexes were calculated from the fatty acid composition: Saturated Fatty Acids (SFA)= Σ % of saturated fatty acids; Unsaturated Fatty Acids (UFA)= Σ % unsaturated fatty acids; Monounsaturated Fatty Acids (MUFA)= Σ % of monoenoic fatty acids; Polyunsaturated n-3 Fatty Acids (PUFAn-3)= Σ % of polyunsaturated fatty acids n-3 serie; Polyunsaturated n-6 Fatty Acids (PUFAn-6)= Σ % of polyunsaturated fatty acids n-6 serie; Average Chain Length (ACL)= [(Σ %Total14 x 14) + …+ (Σ %Total n x n)]/100 (n= carbon atom number); Peroxidizability index (PI) = [(Σ mol% Monoenoic x 0.025) + (Σ mol% Dienoic x 1) + (Σ mol% Trienoic x 2) + (Σ mol% Tetraenoic x 4) + (Σ mol% Pentaenoic x 6) + (Σ mol% Hexaenoic x 8)].

LC-TOF based lipidome analyses

Briefly, lipid samples, extracted as above, after drying and reconstitution with mobile phase (see below) were injected into a reversed-phase chromatography system, using a Zorbax C18 column (150 x 0.5 mm x 5 μm) at a flow rate of 8 μl/min in a LC system consisting of a capillary pump (Agilent 1200). Buffer A was water with 0.1% formic acid, and buffer B was acetonitrile with 0.1% formic acid. The column was equilibrated in 5% B and the gradient was 5%--95%B over 50 min. The eluant was directed to an ESI source, with a nebulizer gas flow of 15 l/min at 300º C with a capillary voltage of 3500 V operating in positive mode, with a dual spray for reference mass (m/z 121.05087 and m/z 922.0098). The instrument was calibrated immediately prior to the test. For the data analysis with the Agilent MassHunter Profiling software, five repeated injections of each extract sample were measured by the same LC/MS method. The acquired MS data were extracted by the Molecular Feature Extractor in the Agilent MassHunter Qualitative software. In this process, the identified ions were clustered to “Molecular features” comprising different isotopic distribution and adducts from the same molecule. The obtained files were grouped according to the samples into two respective groups (control samples *vs* Cu^++^ treated samples or control hypercholesterolemic hamsters *vs* hypercholesterolemic phenolic-enriched vegetal extract supplemented hamsters) and loaded into the Agilent MassHunter Profiling software. For the differential analysis of both groups the features of each group were compared using Student’s T Test. The resulting masses were searched for in the Lipid Maps Databases (http://www.lipidmaps.org/tools/index.html), and among 73 differential molecular features found, 17 were significantly increased (p<0.01) over values found in control incubations of LDL (being the remaining 56 diminished). Among those, the three most abundant had masses compatible with 1-palmitoyllysophosphatidylcholine –PLPC- (measured mass: 495.3328, theoretical mass: 495.3325), 1-stearoyl-sn-glycero-3-phosphocholine –SGPC- (measured mass: 523.364, theoretical mass: 523.3638) and 1-oleoylglycerophosphocholine –OGPC- (measured mass: 521.3476, theoretical mass: 521.3481).

*Cell viability*

HMEC cell line (endothelial cell), kindly donated by Dr. A. Negre-Salvayre (INSERM, Toulouse, France) were cultured in 100mm plates with high glucose DMEM containing 10% FBS until they were 70-80% confluent. Afterwards, the cells were harvested, counted and seeded in 96-well microplates (25,000 cells/well) with the same culture medium, leaving a strip of wells free of cells to be used as a blank. The cells were immediately centrifuged at 1000 rpm for 4 min, without stop brake, in order to obtain a fast and homogeneous attachment to the bottom of the wells. After 6 h, the cell culture medium was removed and serum-free DMEM was added to the cells. One hour later, the culture medium was removed again and 100 μl of serum-free DMEM containing non-oxidized LDL, oxLDL, oxLDL in the presence of phenolic acids or the vehicles alone, was added to each well. The final concentration of LDL in all conditions was 200 μg/ml. Eighteen hours later, the culture medium was removed and 100 μl of PBS was added to each well in order to avoid possible interferences, immediately before determining viability. The effect of phenolic compounds on the cellular capacity to withstand tert-butylhydroperoxide (t-BOOH) challenge was measured. The HepG2 cells were serum starved (0.5% FCS) in the presence of selected phenolics (5 µM) for 8 h. The cells were then challenged with 200 µM t-BOOH as peroxide donor, and 2 h later, cell viability was estimated with the 3-(4,5-Dimethylthiazol-2-yl)-2,5-diphenyltetrazolium bromide (MTT) test, according manufacturer instructions.

Supplemental Literature Cited

1. Pamplona R, Dalfo E, Ayala V, Bellmunt MJ, Prat J, Ferrer I, Portero-Otin M. Proteins in human brain cortex are modified by oxidation, glycoxidation, and lipoxidation. effects of alzheimer disease and identification of lipoxidation targets. J Biol Chem. 2005;280:21522-30.
