# Supplementary material for: Plant-Derived Phenolics Inhibit the Accrual of Structurally Characterised Protein and Lipid Oxidative Modifications
Source: PLoS One. 2012 Aug 29;7(8):e43308. doi: 10.1371/journal.pone.0043308 (PMC3430685; doi:10.1371/journal.pone.0043308)
Supplement: Table S1 — (DOCX) [file pone.0043308.s007.docx]

**Supplemental Table 1**

|  | Control | Polyphenol extract | P (By Student’s T test) |
| --- | --- | --- | --- |
| Final weight (g) | 118.12±7.6 | 114.18±8.6 | 0.062 |
| Total cholesterol (mg/dL) | \| 455,0 ± 36,39 \| \| --- \| | 421,9 ± 12,07 | 0.108 |
| LDL-Cholesterol (mg/dL) | 403,8 ± 50,57 | 345,9 ± 44,09 | 0.4 |
| HDL-Cholesterol (mg/dL) | 327,7 ± 17,63 | 305,4 ± 10,03 | 0.27 |
| Triacilglyceride (mg/dL) | 225,0 ± 35,95 | 142,6 ± 9,492 | 0.051 |
| Alkaline phosphatase  (U/L) | 54.07±18.3 | 47.63±6.6 | 0.56 |

Shown is mean±S.E.M (n=6)
